# EVIDENCE-BASED REVIEW & RECOMMENDATIONS FROM THE BRAZILIAN SOCIETY OF SHOULDER AND ELBOW SURGERY: HYALURONIC ACID VERSUS CORTICOSTEROID INJECTIONS FOR SHOULDER OSTEOARTHRITIS

**DOI:** 10.1590/1413-785220263401e306100

**Published:** 2026-08-31

**Authors:** Paulo Henrique Schmidt Lara, Paulo Santoro Belangero, Márcio Schiefer, Leonardo Zanesco, Marcos Rassi Fernandes, Joel Murachovsky, João Felipe de Medeiros, Eduardo Angeli Malavolta

**Affiliations:** 1 Escola Paulista de Medicina, UNIFESP, Sao Paulo, SP, Brazil.; 2 Instituto Nacional de Traumatologia e Ortopedia Jamil Haddad (INTO), Rio de Janeiro, RJ, Brazil.; 3 Universidade de Sao Paulo, Faculdade de Medicina, Hospital das Clinicas (HCFMUSP), Sao Paulo, SP, Brazil.; 4 Universidade Federal de Goias, Faculdade de Medicina, Goiania, GO, Brazil.; 5 Faculdade de Medicina do ABC, Santo Andre, SP, Brazil.; 6 Universidade Federal do Rio Grande do Norte, Natal, RN, Brazil.

**Keywords:** Osteoarthritis, Shoulder, Corticosteroid, Hyaluronic Acid, Osteoartrite, Ombro, Corticoide, Ácido Hialurônico

## Abstract

**Introduction:**

Shoulder osteoarthritis is an inflammatory and degenerative condition affecting the joint between the humeral head and the glenoid. It is estimated to affect up to 20% of patients aged 60 or older. In our setting, the use of hyaluronic acid is becoming increasingly employed for the treatment of shoulder osteoarthritis. Therefore, this consensus aims to review the current literature to determine whether treatment with hyaluronic acid infiltration is more effective than corticosteroid infiltration in terms of pain outcome for the treatment of shoulder osteoarthritis in adults.

**Methods:**

A systematic review of the literature was conducted in the Pubmed, Embase, Cochrane Library and BVS databases up to November 2025, aiming to evaluate whether intra-articular injection with hyaluronic acid is more effective than with corticosteroids for the treatment of shoulder osteoarthritis in adults.

**Results:**

The systematic review concluded that hyaluronic acid infiltration is more effective than corticosteroid infiltration for pain and function outcomes, mainly after six months post-infiltration.

**Conclusion:**

There is evidence, albeit of very low quality, demonstrating that hyaluronic acid injections are more effective than corticosteroid injections for the treatment of shoulder osteoarthritis, with the best results for both treatment modalities occurring in mild to moderate cases of shoulder osteoarthritis. ***Level of evidence II; Systematic Review.***

## RESUMO

**Introdução:**

A osteoartrose do ombro é uma condição inflamatória e degenerativa que acomete a articulação entre a cabeça umeral e a glenoide. Estima-se que afete até 20% dos pacientes com 60 anos ou mais. Em nosso meio, o uso do ácido hialurônico tem se tornado cada vez mais frequente no tratamento da osteoartrose do ombro. Assim, o presente consenso tem como objetivo revisar a literatura atual para avaliar se a infiltração com ácido hialurônico é mais efetiva do que a infiltração com corticosteroide em termos de desfecho de dor no tratamento da osteoartrose do ombro em adultos.

**Métodos:**

Foi realizada uma revisão sistemática da literatura nas bases de dados PubMed, Embase, Cochrane Library e BVS até novembro de 2025, com o objetivo de avaliar se a injeção intra-articular de ácido hialurônico é mais eficaz do que a de corticosteroide para o tratamento da osteoartrose do ombro em adultos.

**Resultados:**

A revisão sistemática concluiu que a infiltração com ácido hialurônico é mais efetiva do que a infiltração com corticosteroide para os desfechos de dor e função, principalmente após seis meses da infiltração.

**Conclusão:**

Existe evidência, embora de muito baixa qualidade, de que a infiltração com ácido hialurônico é mais efetiva do que a infiltração com corticosteroide para o tratamento da osteoartrose do ombro, sendo que os melhores resultados para ambas as modalidades ocorrem em casos leves a moderados. ***Nível de evidência II; Revisão sistemática.***

## RECOMMENDATION

Regarding the outcome of pain, there is very low-quality evidence demonstrating that intra-articular injection with hyaluronic acid is more effective than intra-articular injection with corticosteroids for the treatment of shoulder osteoarthritis in adults. This effectiveness is greater after 3 months of treatment.

The SBCOC Consensus Project recommends that surgeons individually assess the characteristics of each patient and their condition to make the best treatment decision. Due to the potential superiority of hyaluronic acid in terms of duration and pain control, especially in mild to moderate cases, we recommend hyaluronic acid as the preferred alternative.

## INTRODUCTION

Shoulder osteoarthritis is an inflammatory and degenerative disease that affects the joint between the humeral head and the glenoid.^1,2^ It is estimated to affect between 16% and 20% of patients aged 60 or older, although only 5% to 7% are clinically symptomatic.^3^ Some prevalence studies have indicated that this disease affects more women than men.^4^

Medical literature states that we should use injections to treat shoulder osteoarthritis only when conservative treatment (analgesics, anti-inflammatories, physiotherapy, acupuncture, among others) has failed and when shoulder pain and functional deficit are of moderate to high intensity. The main objectives of this treatment are to improve pain, function, and the possible postponement of glenohumeral arthroplasty.^2,5^

Corticosteroid infiltration theoretically has an anti-inflammatory and analgesic effect, improving symptoms in the short term (up to 8 weeks); however, these effects are not maintained in the short, medium, and long term (above 3 to 6 months). The main complications of this treatment include pain after infiltration, skin atrophy, tendon degeneration and risk of rupture, as well as possible systemic complications.^6,7^

In theory, hyaluronic acid infiltration improves local viscoelasticity, has an analgesic effect, and can stimulate tissue repair and healing. According to the literature, it improves function and pain in the short and medium term (up to 12 months), being reported as superior to corticosteroids in some studies. Complications may include foreign body reactions, infections, allergies, increased pain, among others. However, its theoretical advantages include: fewer local adverse effects compared to corticosteroids, no known systemic reactions, and reduced need for repeated infiltrations.^7–10^

In our setting, the use of hyaluronic acid is becoming increasingly frequent and increasingly employed for the treatment of shoulder osteoarthritis. Therefore, this consensus aims to review the current literature to assess whether treatment with hyaluronic acid infiltration is more effective than corticosteroid infiltration in terms of pain outcome for the treatment of shoulder osteoarthritis in adults.^1,2,10^

### Research Question

Is intra-articular injection with hyaluronic acid more effective than intra-articular injection with corticosteroids for the treatment of shoulder osteoarthritis in adults?

## METHODS

- Databases searched: Pubmed, Embase, Cochrane Library and BVS.
- Descriptors: "Shoulder Joint" [Mesh] OR (Joint, Shoulder) OR (Joints, Shoulder) OR (Shoulder Joints) OR (Glenohumeral Joint) AND "Hyaluronic Acid" [Mesh] OR (Acid, Hyaluronic) OR (Amvisc) OR (Luronit) OR (Hyvisc) OR (Hyaluronan) AND "Adrenal Cortex Hormones" [Mesh] OR (Hormones, Adrenal Cortex) OR (Corticoid) OR (Adrenal Cortex Hormone) OR (Cortex Hormone, Adrenal) AND "Injections, Intra-Articular" [Mesh] OR (Injection, Intra-Articular) OR (Intra-Articular Injection) OR (Intra Articular Injection) OR (Articular Injection, Intra) OR (Articular Injections, Intra) OR (Injection, Intra Articular) OR (Injections, Intra Articular) OR (Intra Articular Injections) OR (Intraarticular Injection) OR (Injection, Intraarticular) (Appendix 1).
- Search period: up to November 2025
- Study selection: updated systematic reviews (SRs) with adequate methodological quality were prioritized. In the absence of SRs, randomized clinical trials (RCTs) were considered, provided that methodological quality had been assessed.

## RESULTS

### Search and study selection

The search located 160 references (56 PubMed, 37 Embase, 41 Cochrane Library, and 26 BVS Regional Portal). Of these, two studies were considered systematic reviews by their primary authors, but both had biases that made their conclusions unsuitable for this consensus.

The review by Colen et al.^11^ (which included any infiltration performed for the treatment of shoulder osteoarthritis) included several types of primary studies (prospective, retrospective, case series, among others) and, therefore, was not considered for our evaluation, as it was a literature review and not a level 1 systematic review.^11^

In November 2025, Migliorini et al.^12^ published a work also titled as a systematic review that included any type of infiltration for the treatment of shoulder osteoarthritis; however, it included several types of primary studies (retrospective cohorts, cohorts, randomized clinical trials), which also prevented our inclusion of this study in our consensus.^12^

**Figure 1:**
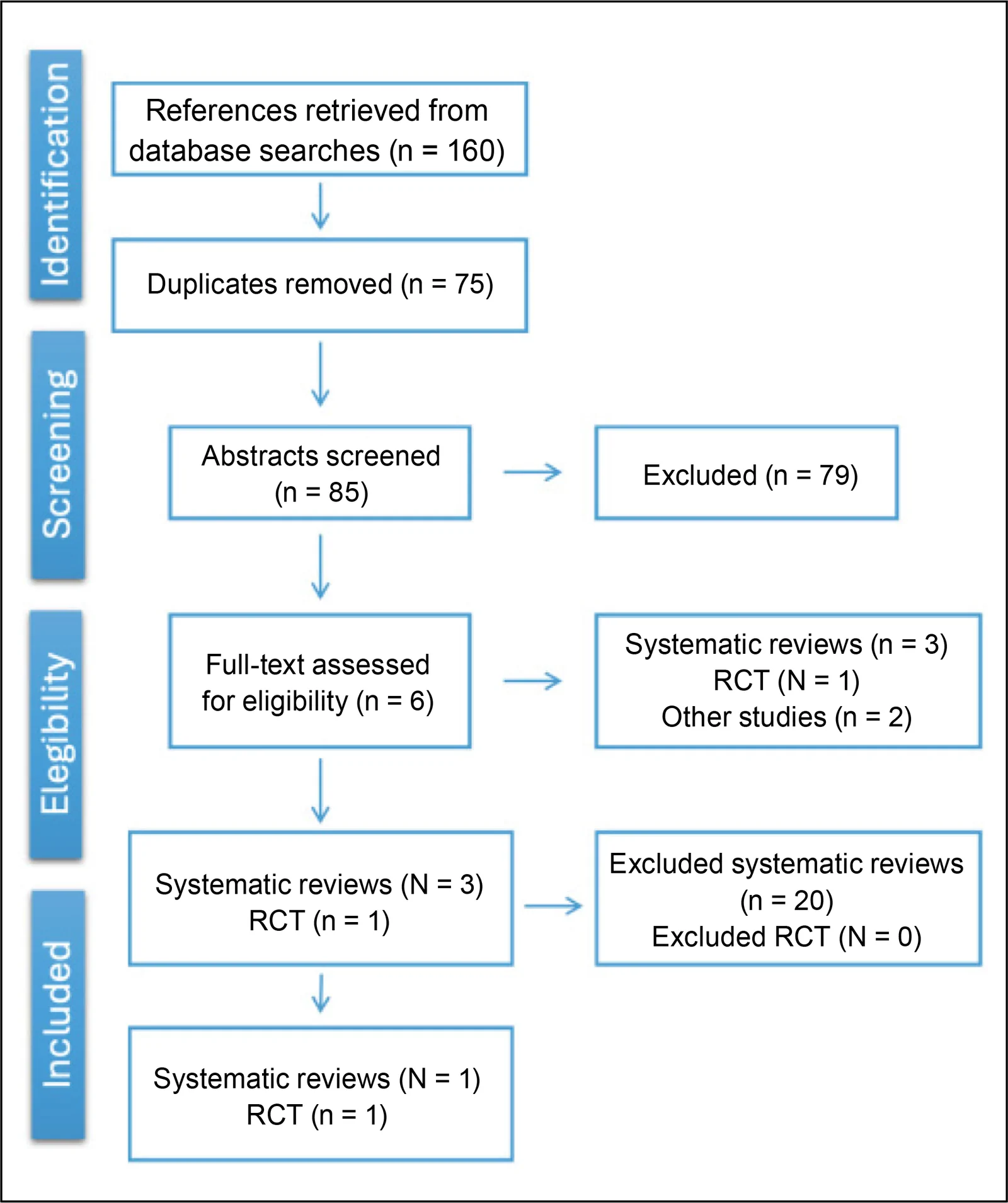
Flowchart of the literature search.

Other studies were also excluded because they were not systematic reviews, randomized clinical trials, or did not address the topic of shoulder osteoarthritis.^13,14^ (Figure 1)

### Included Studies

1 systematic review and 1 randomized clinical trial were included.

The systematic review by Familiari et al.^15^ included only 2 randomized clinical trials that compared hyaluronic acid infiltration with corticosteroids. Thus, with a small sample of 154 participants, the systematic review concluded that hyaluronic acid infiltration is more effective than corticosteroid infiltration for pain and function outcomes, mainly after 6 months post-infiltration. This review included 2 studies: Merolla et al.^16^ and Tortato et al.^17^, which were supposedly randomized clinical trials, but after reading, it was found that the work by Merolla et al.^16^ was a retrospective cohort study, which caused a significant bias in the systematic review.

The randomized clinical trial by Tortato et al.^17^ was conducted in Brazil and compared the use of hyaluronate GF 20 with triamcinolone. In a sample of 77 patients, the outcomes pain, range of motion, Constant score, modified UCLA score, and SPADI index were evaluated. Regarding the outcome pain, it was assessed using the VAS (Visual Analogue Scale), and it was reported that 76% of patients who received hyaluronic acid injections showed improvement in pain after 1 month, and 63% showed improvement after 6 months compared to the initial assessment. Patients classified as having "non-severe" arthritis obtained better results than those classified as having "severe" arthritis. And, the hyaluronic acid group had better results mainly after the third month. The authors concluded that: "Both injections reduced pain and increased patient satisfaction. Results tend to be better and more lasting in patients receiving hyaluronic acid."

The general characteristics of the evaluated studies and the quality of evidence according to GRADE^18,19^ can be observed in Tables 1 and 2, respectively.

**Table 1:** Characteristics of the included studies.

Included studies	Type of study	Included studies (types of study)	Casuistry	Previously published protocol
Familiari et al. (2023)	Systematic review	2 (1 RCT, 1 cohort)	154	Yes
Tortato et a1.^17^ (2022)	Randomized clinical trial (RCT)	---	77	No, but it was registered

**Table 2:** Quality of evidence (GRADE): Hyaluronic acid infiltration versus corticosteroid.

Outcome	Studies (n)	Evidence quality
Pain	2 studies (154)	Very low^1^,^2^,^3^
Shoulder function (varied scores)	2 studies (143)	Very low^1^,^2^,^3^

1 Limited sample size;

2 Imprecision (large confidence interval);

3 Risk of bias in included studies.

4 Lack of prior protocol.

Note: GRADE (Grading of Recommendations Assessment, Development and Evaluation) is a tool used to assess and measure the quality of evidence. It is based on 5 pillars: Risk of bias, Imprecision, Inconsistency, Indirect assessment of outcomes, and Publication bias. With this, the quality of evidence is classified as: very low, low, moderate, and high quality.^18,19^

## CONCLUSIONS

There is evidence, albeit of very low quality, demonstrating that hyaluronic acid injections are more effective than corticosteroid injections for the treatment of shoulder osteoarthritis, with the best results for both treatment modalities occurring in mild to moderate cases of shoulder osteoarthritis.

### Implications for Research

More randomized clinical trials with improved methodological quality are needed to compare hyaluronic acid injections versus corticosteroid injections for the treatment of shoulder osteoarthritis.

## Data Availability

The data underlying this study are available within the article.

## Appendix 1 Search Strategies Across Databases.

**Table:**

**PUBMED**
#1"Shoulder Joint"[Mesh] OR (Joint, Shoulder) OR (Joints, Shoulder) OR (Shoulder Joints) OR (Glenohumeral Joint) OR (Glenohumeral Joints) OR (Joint, Glenohumeral) OR (Joints, Glenohumeral) OR (Glenoid Labrum) OR (Labrum, Glenoid)OR (Glenohumeral Joint) OR (Shoulder Arthritis) OR (Glenohumeral Arthritis)
#2 "Hyaluronic Acid"[Mesh] OR (Acid, Hyaluronic) OR (Amvisc) OR (Luronit) OR (Hyvisc) OR (Hyaluronan) OR (Sodium Hyaluronate) OR (Hyaluronate, Sodium) OR (Hyaluronate Sodium) OR (Healon) OR (Etamucine) OR (Amo Vitrax) OR (Vitrax, Amo) OR (Biolon) OR Hyaluronate OR (Sodium Hyaluronate) OR Viscosupplementation
#3 "Adrenal Cortex Hormones"[Mesh] OR (Hormones, Adrenal Cortex) OR (Corticoid) OR (Adrenal Cortex Hormone) OR (Cortex Hormone, Adrenal) OR (Hormone, Adrenal Cortex) OR (Corticosteroid) OR (Corticoids) OR (Corticosteroids) OR Corticosteroids OR (Steroid Injections) OR Triamcinolone OR Methylprednisolone
#4 "Injections, Intra-Articular"[Mesh] OR (Injection, Intra-Articular) OR (Intra-Articular Injection) OR (Intra Articular Injection) OR (Articular Injection, Intra) OR (Articular Injections, Intra) OR (Injection, Intra Articular) OR (Injections, Intra Articular) OR (Intra Articular Injections) OR (Intraarticular Injection) OR (Injection, Intraarticular) OR (Intra-Articular Injections) OR (Injections, Intraarticular) OR (Intraarticular Injections) OR (Intra-articular Injection) OR (Joint Injection)
#5"Pain"[MeSH] OR (Pain Management) OR (Pain Relief) OR (Pain Measurement)
#5 #1 AND #2 AND #3 AND #4
Filters applied: Clinical Trial; Meta-Analysis; Randomized Controlled Trial; Review; Systematic Review = 56

**Table:**

**COCHRANE**
#1(Shoulder Joint) OR (Joint Shoulder) OR (Shoulder Joints) OR (Glenohumeral Joint) OR (Glenohumeral Joints) OR (Joint Glenohumeral) OR (Joints Glenohumeral) OR (Glenoid Labrum) OR (Labrum Glenoid) OR (Glenohumeral Joint) OR (Shoulder Arthritis) OR (Glenohumeral Arthritis)
#2 (Hyaluronic Acid) OR (Acid Hyaluronic) OR (Amvisc) OR (Luronit) OR (Hyvisc) OR (Hyaluronan) OR (Sodium Hyaluronate) OR (Hyaluronate Sodium) OR (Hyaluronate Sodium) OR (Healon) OR (Etamucine) OR (Amo Vitrax) OR (Vitrax Amo) OR (Biolon) OR Hyaluronate OR (Sodium Hyaluronate) OR Viscosupplementation
#3 (Adrenal Cortex Hormones) OR (Hormones Adrenal Cortex) OR (Corticoid) OR (Adrenal Cortex Hormone) OR (Cortex Hormone Adrenal) OR (Hormone Adrenal Cortex) OR (Corticosteroid) OR (Corticoids) OR (Corticosteroids) OR Corticosteroids OR (Steroid Injections) OR Triamcinolone OR Methylprednisolone
#4 (Injections Intra-Articular) OR (Injection Intra-Articular) OR (Intra-Articular Injection) OR (Intra Articular Injection) OR (Articular Injection Intra) OR (Articular Injections Intra) OR (Injection Intra Articular) OR (Injections Intra Articular) OR (Intra Articular Injections) OR (Intraarticular Injection) OR (Injection Intraarticular) OR (Intra-Articular Injections) OR (Injections, Intraarticular) OR (Intraarticular Injections) OR (Intra-articular Injection) OR (Joint Injection)
#5 #1 AND #2 AND #3 AND #4 = 41

**Table:**

**EMBASE**
#1 ‘shoulder joint’/exp OR ‘articulatio glenohumeralis’ OR ‘articulatio humeri’ OR ‘gleno-humeral joint’ OR ‘glenohumeral joint’ OR ‘humeral joint’ OR ‘humero-scapular joint’ OR ‘humeroscapular joint’ OR ‘joint, glenohumeral’ OR ‘joint, humeroscapular’ OR ‘scapulo humeral joint’ OR ‘scapulohumeral joint’ OR ‘shoulder (joint)’ OR ‘shoulder joint’
#2 ‘hyaluronic acid’/exp OR ‘adant’ OR ‘adant dispo’ OR ‘amo vitrax’ OR ‘amvisc’ OR ‘amvisc plus’ OR ‘arthrease’ OR ‘artz’ OR ‘biolon’ OR ‘bionect’ OR ‘clearvisc’ OR ‘duovisc’ OR ‘durolane’ OR ‘eyecon’ OR ‘go-on (drug)’ OR ‘halonix’ OR ‘healon’ OR ‘healon gv’ OR ‘healon yellow’ OR ‘healon5’ OR ‘healonid’ OR ‘hialid’ OR ‘hyalcon’ OR ‘hyalein’ OR ‘hyalgal’ OR ‘hyalgan’ OR ‘hyalovet’ OR ‘hyalubrix’ OR ‘hyaluronan’ OR ‘hyaluronate’ OR ‘hyaluronate sodium’ OR ‘hyaluronic acid’ OR ‘hyaluronic acid component’ OR ‘hyladerm’ OR ‘hylaform’ OR ‘hylan g f 20’ OR ‘hylan g-f 20’ OR ‘hylartin v’ OR ‘hylo-comod’ OR ‘hylumed’ OR ‘hyruan’ OR ‘ialugen’ OR ‘juvederm’ OR ‘lagricel ofteno’ OR ‘laservis’ OR ‘me 3710’ OR ‘monovisc’ OR ‘na hylan’ OR ‘na-hylan’ OR ‘nrd 101’ OR ‘nrd101’ OR ‘ophthalin’ OR ‘ophthalin plus’ OR ‘orthovisc’ OR ‘ostenil’ OR ‘perlane’ OR ‘potassium hyaluronate’ OR ‘provisc’ OR ‘radiaplexrx’ OR ‘restylane’ OR ‘restylane lyft’ OR ‘si 4402’ OR ‘sinovial’ OR ‘sl 1010’ OR ‘sodium hyaluronate’ OR ‘sperm select’ OR ‘supartz’ OR ‘suplasyn’ OR ‘synocrom’ OR ‘synojoynt’ OR ‘synvisc’ OR ‘teosyal’ OR ‘triluron’ OR ‘unihylon’ OR ‘viscoseal’ OR ‘vismed’ OR ‘vitrax’
#3 ‘corticosteroid’/exp OR ‘adrenal cortex hormone’ OR ‘adrenal cortex hormones’ OR ‘adrenal cortical hormone’ OR ‘adrenal cortical hormones’ OR ‘adrenal cortical steroid’ OR ‘adrenal steroid’ OR ‘adrenal steroid hormone’ OR ‘adreno cortical steroid’ OR ‘adreno corticosteroid’ OR ‘adrenocortical hormone’ OR ‘adrenocortical steroid’ OR ‘adrenocorticosteroid’ OR ‘cortical steroid’ OR ‘cortico steroid’ OR ‘corticoid’ OR ‘corticosteroid’ OR ‘corticosteroid agent’ OR ‘corticosteroid calcium’ OR ‘corticosteroid hormone’ OR ‘corticosteroids’ OR ‘corticosteroids, inhalation’ OR ‘corticosteroids, ophthalmic’ OR ‘corticosteroids, otic’ OR ‘corticosteroids, systemic’ OR ‘corticosteroids, topical’ OR ‘dermocorticosteroid’ OR ‘fluorinated corticosteroid’
#4 ‘intraarticular drug administration’/exp OR ‘drug administration, intraarticular’ OR ‘injection, intraarticular’ OR ‘injections, intra-articular’ OR ‘intra-articular administration’ OR ‘intra-articular delivery’ OR ‘intra-articular drug administration’ OR ‘intra-articular drug delivery’ OR ‘intra-articular infusion’ OR ‘intra-articular injection’ OR ‘intra-articular injections’ OR ‘intra-articular medication’ OR ‘intraarticular treatment’ OR ‘intraarticular administration’ OR ‘intraarticular delivery’ OR ‘intraarticular drug administration’ OR ‘intraarticular infusion’ OR ‘intraarticular injection’ OR ‘intraarticular medication’ OR ‘intraarticular treatment’ OR ‘intracoxal drug administration’ OR ‘joint infusion’ OR ‘joint injection’ OR ‘treatment, intraarticular’
#5 #1 AND #2 AND #3 AND #4 = 37

**Table:**

**PORTAL REGIONAL BVS**
#1 MH:"Articulação do Ombro" OR (Shoulder Joint) OR (Articulação Glenoumeral) OR (Articulações dos Ombros) OR (Ligamento Glenoide Umeral) OR (Lábio Glenoidal) OR MH:A02.835.583.748$ OR (Articulação do Ombro) OR (Artrite Glenoumeral)
#2 MH:"Ácido Hialurônico" OR (Hyaluronic Acid) OR (Hialuronan) OR MH:D09.698.373.475$ OR (Ácido Hialurônico) OR (Hialuronato de Sódio) OR (Viscossuplementação)
#3 MH:"Corticosteroides" OR (Adrenal Cortex Hormones) OR (Corticoesteroides) OR (Corticoide) OR (Corticoides) OR (Corticosteroide) OR (Hormônio Adrenocortical) OR (Hormônio Córtico-Adrenal) OR (Hormônio do Córtex Ad-Renal) OR (Hormônio do Córtex Adrenal) OR (Hormônio do Córtex das Ad-Renais) OR (Hormônio do Córtex das Glândulas Ad-Renais) OR (Hormônio do Córtex das Glândulas Suprarrenais) OR (Hormônios Adrenocorticais) OR (Hormônios Córtico-Adrenais) OR (Hormônios do Córtex Ad-Renal) OR (Hormônios do Córtex Suprarrenal) OR (Hormônios do Córtex das Ad-Renais) OR (Hormônios do Córtex das Glândulas Ad-Renais) OR (Hormônios do Córtex das Glândulas Suprarrenais) OR (Hormônios do Córtex das Suprarrenais) OR MH:D06.472.040$ OR(Hormônios do Córtex Adrenal) OR Corticosteroides OR (Triancinolona) OR (Metilprednisolona)
#4 MH:"Injeções Intra-Articulares" OR (Injections, Intra-Articular) OR (Injeção Intra-Articular) OR MH:E02.319.267.530.380$ OR (Injeções IntraArticulares) OR (Infiltração articular)
#5 #1 AND #2 AND #3 AND #4 = 26
MEDLINE (22)
WPRIM (Pacífico Ocidental) (2)
IBECS (1)
LILACS (1)
